# Supplementary material for: Anti-Metastatic Effects of Standardized Polysaccharide Fraction from Diospyros kaki Leaves via GSK3β/β-Catenin and JNK Inactivation in Human Colon Cancer Cells
Source: Polymers (Basel). 2024 May 3;16(9):1275. doi: 10.3390/polym16091275 (PMC11085380; doi:10.3390/polym16091275)
Supplement: Supplementary file 1 [file polymers-16-01275-s001.zip › polymers-2920409 Supplementary.pdf]

Supplementary Data

Supplementary Table S1. Antibodies used in the western blot analysis

Supplier	Location	Antibodies
Santa Cruz Biotechnology	Santa Cruz, CA, USA	MMP-9 (sc-21733), TIMP-1 (sc-5538), β-catenin (sc-376841), GSK3β (sc-81462), p-c-Jun (sc-822), c-Jun (sc-74543), c-Fos (sc-253), p-JNK (sc-6254), p-ERK (sc-7383), JNK (sc-7345), ERK (sc-94), p65 (sc-8008), and β-actin (sc-81178)
Cell signaling Technology	Danvers, MA, USA	MMP-2 (#4022), p-GSK3β (#5558), p-c-Fos (#5348), p-p65 (#3033), p38 (#9211), and p-p38 (#9215)

Supplementary Table S2. Oligonucleotide primer sequences for qRT-PCR

Gene	Forward	Reverse
E-cadherin	TCTTCTCCGCCTCCTTCTTC	AATTCCTGCCATTCTGGGGA
N-cadherin	TGAGCCTGAAGCCAACCTTA	AGGTCCCCTGGAGTTTTCTG
Vimentin	AGCTAACCAACGACAAAGCC	TCCACTTTGCGTTCAAGGTC
TIMP-1	CTGGAAAACTGCAGGATGGA	GTGCATTCCTCACAGCCAAC
MMP-2	ACCGCGACAAGAAGTATGGC	CCACTTGCGGTCATCATCGT
MMP-9	CGATGACGAGTTGTGGTCCC	TCGTAGTTGGCCGTGGTACT
β-actin	ATCACTATTGGCAACGAGCG	TCAGCAATGCCTGGGTACAT
